# Survey of Industrial, Agricultural, and Medical Applications of Radiometric Gauging and Process Control

**DOI:** 10.6028/jres.095.053

**Published:** 1990

**Authors:** J. H. Hubbell

**Affiliations:** National Institute of Standards and Technology, Gaithersburg, MD 20899

**Keywords:** albedo, density, electrons, gamma rays, gages, gauges, metrology, moisture, neutrons, radiation, radiometric, thickness, transmission, x rays

## Abstract

Photon and particle radiations (gamma rays, x rays, brems-strahlung, electrons and other charged particles, and neutrons) from radioactive isotopes, x-ray tubes, and accelerators are now widely used in gauging, production control, and other monitoring and metrology devices where avoidance of mechanical contact is desirable. The general principles of radiation gauges, which rely on detection of radiation transmitted by the sample, or on detection of scattered or other secondary radiations produced in the sample, are discussed. Examples of such devices currently used or at least shown to be feasible in industrial, transportation, building, mining, agricultural, medical, and other metrology situations are presented, drawing from a total of 146 selected technical and review paper reference sources here cited.

## 1. Introduction

Radiometric gauging is a powerful and generally noninvasive, nondestructive metrology tool used in a variety of industrial, agricultural, and medical human enterprises. Not only the industrialized nations, but also many less-developed countries have found this “high tech” (radiation physics) methodology to be readily adaptable to regional metrology needs following various indigenous original developments.

The radiations usually employed in radiometric gauging and control devices are photons (x rays, gamma rays, bremsstrahlung), electron beams (beta rays), neutrons, and, more rarely, alpha and other heavy charged particles. Types of gauges include transmission gauges, albedo (backscatter) gauges, x-ray fluorescence gauges, and other devices employing a variety of radiation physics, atomic physics, and nuclear physics principles, sometimes rather exotic such as the Mossbauer effect. Radiometric gauging and control devices have been reviewed by a number of authors, including, for example, Taylor [1], Palmer [2], Bock [3], Snow and Morris [4], Hubbell [5,6], and Clayton [7]. A review specializing in geology, mining, and metallurgy has been given by Kartashev [8], and on radiometric gauging in Portugal by Salgado et al. **[9].** The International Organization for Standardization (ISO) [10] has developed and issued a Standard for design and application of radionuclide gauges, which is also a good source of information.

## 2. General Principles of Radiometric Gauging and Control

Radiation gauges necessarily involve a radiation source, surrounded by appropriate shielding to minimize any health hazard, plus usually a collimator to confine the radiation to a narrow beam as it impinges on the material sample. The other essential element of the gauge is a radiation detector, usually energy-selective, and also collimated and shielded, and associated electronics, as described, for example, in the level-gauging paper by Heier [11]. General approaches to the design, optimization, calibration, and error sources of both static (sample properties remain constant during measurement period) and dynamic (sample properties change during measurement) radiation gauges have been given by Urbanski [12,13], Henderson and McGhee [14], Notea and Segal [15,16], Zav’yalkin and Osipov [17], and Kasi [18].

In transmission (attenuation) gauges, as recently discussed by Bernhardt [19] and Oyedele [20,21] in the context of sheet-material production thickness control, the detector is located so as to directly “see” the collimated beam of radiation with intensity *I*_0_ from the source. The sample is then interposed in the beam with surfaces normal to the beam direction, resulting in attenuation of the detector signal to an intensity *I*, for photons exponentially related to the sample thickness t (in mass per unit area) according to

$$I/I_{0}=\exp(-(\mu /\rho )t)$$ (1)

where *μ*/*ρ* (in area per unit mass) is the mass attenuation coefficient for the sample material. Hence, for example, the mass per unit area thickness *t* of a sample can be determined as

$$t=[\ln(I_{0}/I)]/(\mu /\rho )$$ (2)

from measurements of photon (x ray or gamma ray) intensities *I*_0_ (no sample in beam) and *I* (sample in beam) and a knowledge of photon *μ*/ρ data from published tables such as those of Hubbell [22], Berger and Hubbell [23], or Cullen et al. [24].

Scatter gauges for density and thickness measurements are also widely used, usually relying on photon Compton scattering, the energy-angle relationship for this process, and the dependence of this process on electron density in the sample, as discussed by Taylor and Kansara [25,26], Pandey [27], Gayer et al. [28], and Zabrodskii [29,30,31]. Although the detector, which may be either on the same or far side of the sample from the radiation source, is usually collimated to restrict the detector view, in some monitoring devices neither the source nor the detector is collimated. When the detector and source are on the same side of the sample, the device is known as an albedo or backscatter gauge. Golikov et al. [32] have described a sheet-collimation gamma-ray albedo gauge for multi-layer structures, and Mohammadi [33] describes both gamma and electron-albedo gauges for measurement of glass container wall thickness.

Although x-ray fluorescence (XRF) is more widely used for chemical analysis than for gauging, XRF is sometimes used as a type of albedo gauge for thickness measurements of thin films and coatings as discussed, e.g., by Saneyoshi et al. [34]. Analogous to XRF but involving atomic nuclei instead of atomic electron shells is the use of neutrons and the resultant gamma rays from inelastic scattering, radiative capture, and other nuclear interactions, for materials monitoring, as discussed by Pekarski [35]. Included in the variety of other radiation gauging and monitoring devices is detection of positron annihilation radiation for determination of mean radius and concentration of micropores in porous materials such as ceramics and powdered alloys as discussed by Semenov [36]. Radiographic determination of thickness variations using film detectors has been discussed by Lahure [37], and a good overview of neutron radiography and gauging can be obtained from the papers in an ASTM conference proceedings edited by Berger [38].

## 3. Radiometric Gauging and Control in In-Plant Industries

Computer aided (or axial) tomography (CAT scanning), first finding its dramatic application in medical diagnostic radiography, is now finding wide and very advantageous use in on-line dimensional, shape, and flaw-detection gauging and production control in in-plant industrial situations, as discussed by Morgan [39], Martz et al. [40], Seshadri et al. [41], and Altukhov et al. [42]. A somewhat simpler large-object flaw-detection device, using a thin fan-beam photon source and a one-dimensional array of silicon detectors has been described, e.g., by Gusev et al. [43].

In in-plant industries, an accurate knowledge of the level of material inside closed tanks is frequently required. Several kinds of radiometric level gauges are in use, including gamma transmission gauges (e.g., Apelblat [44] and Amberger and Heier [45]), gamma albedo gauges (e.g., Ochiana et al. [46]), and neutron albedo level gauges which are particularly well-suited to hydrogenous substances (Mathew et al. [47]). A linear-detection radiometric liquid-level gauge employing an array of sources (e.g., ^137^Cs) has been described by Gläser and Emmelmann [48]. When the material in the tank is radioactive, the level gauge consists of the detector only (Dickstein and Notea [49]), since the sample is also the radiation source.

Techniques for radiometric bulk density measurements of powdered process materials are reviewed by Thyn and Pokorny [50], and for density distribution measurements in dynamic high-temperature systems by Kondic and Lassahn [51]. Gamma-ray transmission measurements can be used in tubing-wall thickness monitoring (e.g., Frevert [52,53]), and gamma albedo tubing coating thickness measurements are described by Kapranov et al. [54]. A number of coating-thickness radiometric measurement techniques are available, including a scheme described by Grupper [55] utilizing a positron emitting radionuclide source, in which the positron absorption in the coating is measured by detecting the annihilation radiation. A more widely-used coating and thin film measurement technique is x-ray fluorescence (XRF) as described, for example, by Salmi et al. [56], Singh et al. [57], Kaushik et al. [58], Luzzi et al. [59], and Kowalska and Urbanski [60]. A related technique for thin film thickness measurement is PIXE (particle-induced x-ray emission) as described by Miranda et al. [61] and compared with RBS (Rutherford backscattering, using ions with energy of the order of 0.5 MeV) by Oliver and Miranda [62].

A microcomputer-based gamma absorption gauge for routine production control of profiled rubber strip, also a beta-absorption monitor control for production of plastic foil in the 20 to 35 *μ*m thickness range, have been described by Tabor et al. [63]. In steel sheet hot rolling, x-ray transmission gauges are now used almost exclusively for roller control to achieve and maintain thickness dimension within desired tolerances as described, e.g., by Petushkov et al. [64] and by Firstov et al. [65]. A neutron gauge for determining acid concentrations in industrial pipelines has been described by Mirowicz and Lis [66], in which the hydrogen content in the acid solution is inferred from the slowing down of fast neutrons from a Pu-Be source and detection of thermal neutrons. For dynamic thickness measurements of liquid films, a tracer technique using technetium 99m, for industrial application, has been described by Stopporka et al. [67]. On-stream analysis of material flowing in pipes, including chemical information, by energy-dispersive x-ray fluorescence, has been discussed by Donhoffer [68].

For two-phase flow within pipes, Oyedele [69,70] has studied the effect of void distributions on void determination using a gamma transmission gauge. Lin et al. [71] have described a pulsed photon activation (PPA) technique for nonintrusive measurements of single- and two-phase flows in a horizontal pipe. For measuring void fraction in a vertical pipe containing a flowing air-water mixture, Wang and Shih [72] describe a technique utilizing a bromine-82 gamma-ray source and a NaI(T1) scintillation detector. Hussein [73] has described a neutron scattering system (“scatterometer”) for measuring the void fraction in a gas-liquid flow, and Hussein and Waller [74] have also incorporated this device into a steam-quality meter in a fluidized bed plant, in which coal is burned with limestone in a “bed” suspended in air in a combuster.

To answer the demand for production quality control of new high-performance reinforced plastics, Entine et al. [75] have developed an x-ray transmission and scattering device for analytical measurement of the glass, graphite, and other fillers used in these plastics. In the fabrication of laser fusion targets, small spherical capsules containing deuterium-tritium, for the Lawrence Livermore National Laboratory (LLNL) Inertial Confinement Fusion (ICF) program, the absorption of liquid into a foam needed to be characterized, which proved to be amenable to an x-ray radiographic (vidicon) technique described by Rikard and Streit [76].

## 4. Radiometric Gauging and Control in Transportation and Building Industries

A current problem in the cargo and passenger air transport industry is the reliability of the fuel quantity gauging (FQG) systems aboard aircraft. Present aircraft FQG systems are based on the old capacitance gauges which sometimes suffer from fouling and electrical noise problems. Singh et al. [77] and Singh [78] have demonstrated the feasibility of a gamma-ray attenuation gauge using a weak ^241^Am (59.5 keV) collimated radiation source and a colinear collimated detector capable of continuously monitoring the fuel quantity in the aircraft tanks to an accuracy of better than 1%.

In the transport industries the density of highway concrete is related to its durability, and a two-channel gamma albedo density gauge is described by Groshev and Zabrodski [79] in which one detector views the highway surface where the gamma beam enters, and the other detector views a scattering volume below the surface. Another gamma albedo gauge, for assessing the density of concrete in both fresh and hardened states, consists of an uncollimated source and detector arrangement (Adil [80]). For measuring the density of the asphalt layer (thin lift) laid down on a repaved highway, a special gamma albedo gauge was developed by Dunn and Hutchinson [81]. A rather interesting example of radiometric gauging in the railway transport industries is gamma-ray albedo examination of railway ties (sleepers) for termite damage (Fookes et al. [82]).

Pipeline transport of solids in a slurry form can be a useful alternative to road and rail transport in terms of exhaust-fume air-pollution, vehicular accidents, and other hazards, as well as possible economic advantages in some cases. In the most usual situation in which the particles are denser than the fluid, for horizontal flow the concentration of solid particles is higher near the bottom of the pipe, resulting in a higher erosion rate in the bottom inside surface of the pipe than in the sides and top. Rohella [83] has demonstrated and described a gamma-ray attenuation device for measuring and monitoring both the chord-average concentration of solids in the flowing slurry, which determines the pipeline capacity, and the concentration gradient which affects the pipeline longevity.

In the building construction industry, an important parameter is the density of rock, soil, and other materials, characterizing a given building site, to be disturbed and to support the building. Henderson and McGhee [84,85] have mathematically modelled a gamma-ray backscattering (albedo) gauge using ^137^Cs (0.66 MeV) or ^60^Co (1.17, 1.33 MeV) sealed “not too powerful” (e.g., 74 MBq) radiation sources for probing near-surface rock and soil densities without the need of the boreholes required in transmission gauging. For examining concrete walls in existing buildings, particularly for the presence, quantity, size, and position of steel reinforcing bars (rebar), and also for voids, Hussein and Whynot [86] and Tuzi and Sato [87] have demonstrated and described Compton-scattering gamma-ray single-scatter albedo probes. Such probes examine the small volume which is the common volume of intersection of the projections of the source collimator and the detector collimator, axially intersecting inside the concrete wall material, which volume can then be characterized by its density and other gamma-ray Compton scattering properties. If these collimator projections inside the concrete wall can be approximated by right circular cylinders of unequal or equal radii, this inter-section volume may be obtained from formulas or a table given by Hubbell [88].

## 5. Radiometric Gauging and Control in the Mineral Industries

In addition to the work by Kartashev [8] mentioned earlier, a good overview of applications of radiometric gauging and control in the mineral industries is the report given by Watt [89] at the Fifth Pacific Basin Nuclear Conference (PBNC-5, 1985) in Seoul. The radiation physics basis for devices for formation lithology logging (well logging) with gamma rays, including Compton scattering and photoelectric absorption, is outlined by Bertozzi et al. [90].

In coal mining, an advance coal-degasification technique is used to remove much of the methane gas trapped in coal seams prior to the mining process in order to reduce the possibility of explosions during mining. In this technique, boreholes approximately 10 cm in diameter are drilled as deep as 600 m into the working faces of the mine. The methane gas permeates through the coal into the boreholes, from which as much as 50% of the methane in the seam can be captured and safely removed. A major obstacle in this technique is that coal seams are in general not straight, requiring periodical removal of the bit and taking of core samples to be sure that the bit has not left the seam. The drill strings used for this process have hydraulically powered drill bits which can be steered slightly into a curved drill path chosen by the operator. To provide steering information to the operator. Entine et al. [91] have developed and demonstrated a CdTe solid-state detector probe, mounted adjacent to the drill tip, which detects the natural radioactivity (usually of uranium, with gamma rays of several hundred keV energy) in the shale outside the seam, and hence the nearness of the drill bit to the seam edge-region where, incidentally, is also found the coal highest in both ash and sulfur content.

On-stream and bulk analysis radiometric devices for probing iron and other ores on moving conveyor belts, using a variety of radiations including gamma rays and both fast and thermal neutrons have been described by Holmes [92] and by Borsaru et al. [93]. A time-of-flight fast-neutron probe useful not only for coal analysis and oil shale assay, but also adaptable to detection of contraband drugs and explosives, has been successfully demonstrated by Gordon and Peters [94].

Production of synthetic crudes is currently receiving some attention, since depletion of natural crudes is effectively irreversible, and because political and now military turmoil has rendered highly uncertain some major sources of imported natural crudes. One of the processes being developed by CANMET (Canada Centre for Mineral and Energy Technology, Ottawa) to produce synthetic crudes by upgrading heavy oils, refinery residua, tar sand bitumen, and coal, is by adding hydrogen to increase the H/C ratio by various techniques, such as hydrocracking, all of which require high temperatures and pressures and close monitoring of the multi-phase hydrodynamic activity inside closed chemical reactors. Liu and Patmore [95] have demonstrated a ^137^Cs gamma-ray attenuation scanning densitometer on-line in a hydrocracking pilot plant, as a noninvasive probe of the H/C ratio enhancement chemical processes.

For determining the ash content, also calcium and iron oxides, in brown coal, a computer controlled probe based on XRF (x-ray fluorescence) and scattering of the low energy x rays from a ^238^Pu source is described by Antoniak et al. [96]. For ash content determination of washed coking coal on a conveyor belt, a dual energy gamma-ray transmission gauge was demonstrated by Gravitis et al. [97] over a 13-month trial period to measure ash contents in the 5%–10% (wt) range differing from chemical assays by 0.3%–0.4%, and related techniques for on-line radiometric analysis and grading in mineral and coal processing are reviewed by Cutmore et al. [98]. An x-ray albedo gauge for determining ash in coal and coke, with correction for moisture content, has been described by Pandey and Prasad [99], and a combination x-ray transmission and albedo gauge “SIRO” (Scientific and Industrial Research Organization) for monitoring solids weight fraction and ash content of coal slurries of variable voidage has been demonstrated by Gravitis et al. [100]. Combination gamma-albedo and neutron-activation gauges have been developed by Holmes et al. [101] for on-stream (on conveyor belts) and bulk (in bins) analysis of iron ores to determine shale content and other information needed for ore grading. Holmes et al. [101] also describe a gauge for determining the iron content in iron ores, in which the ore is irradiated by photons from ^226^Ra, and the 0.511 MeV photons resulting from electron-positron pair production and annihilation are counted, based on principles developed by Sowerby and Ngo [102] and by Millen and Sowerby [103] for measuring ash content in coal.

## 6. Radiometric Gauging and Control in the Agricultural and Forest Industries

Radiometric gauging is used in all stages of the agricultural process, from snowpack profile radioisotopic measurements (e.g., Smith et al. [104]) to radiometric monitoring and control of product packaging such as cottonwool bales for the bandage industry (Tabor et al. [105]). Fishman et al. [106] describe a soil moisture gamma-ray transmission gauge for use as a control unit for automatic irrigation in a field, also as a scanner for developing regional irrigation plans. A gamma-ray backscattering soil density gauge was demonstrated by Pirie et al. [107], and Ertek and Haselberger [108] have developed a gamma multiple-scattering gauge for determining both density and water content in soil. For greater sensitivity to the soil moisture content, Ciftcioglu and Taylor [109] and Ciftcioglu et al. [110] describe a gamma-ray backscatter soil gauge with differential-mode counting. Neutron soil moisture gauges, including an analysis of the size of the region sampled by the device, are described by Kasi and Koskinen [111], Kasi [112], and by Kasi et al. [113]. Wilson [114] presents a parametric study of neutron backscattering soil moisture meters using transport theory.

Schätzler and Kühn [115] have developed a gamma-ray transmission and scanning device to measure and monitor the biomass in a field of growing plants, such as cereal grains, and Kühn [116] also describes a gamma-ray transmission device for monitoring the growth of individual agricultural products such as cabbage heads. In the latter application, the sawtooth growth curve of a cabbage head, from sprout to coleslaw, clearly revealed the biomass increase at night and loss during the day due to evaporation, also that wrapping the cabbage head in clear plastic for a segment of its life had no noticeable effect on this characteristic growth curve.

The moisture content of wheat is a critical parameter for the milling of wheat, and to achieve optimum milling performance it is usual to temper wheat to a set moisture content before milling, best done by using an on-line moisture measuring system to control the addition of water to the wheat. For this purpose a device for measuring both the moisture content and the density of the wheat by simultaneous neutron and gamma-ray transmission (NEUGAT technique) has been demonstrated by Bartle et al. [117], who also examined the merits of three different radiation sources: Am-Be (neutron mean energy 4.5 MeV, gamma-ray highest energies 4.4 MeV), an accelerator-base pulsed source (monoenergetic neutrons 4.5 MeV, gamma-ray mean energy 1.5 MeV), and ^252^Cf (neutron mean energy 2 MeV, gamma-ray mean energy 1 MeV). They concluded that the accelerator source has the highest figure of merit, followed by ^252^Cf and ^241^Am-Be, but that in practice the ^252^Cf source may be preferred because of its simplicity. Another foodstuff monitoring radiometric device, described by Glaser et al. [118], is a two-energy (^241^Am: 60 keV, ^137^Cs: 662 keV) gamma-ray transmission gauge (KRAS-2), originally developed for ash content determination in lignite, to measure the amount of rocks and other mineral material accompanying potatoes on a conveyor belt transporting the potatoes into a storage facility.

In the forest industries, gamma-ray transmission gauges for determination of wood density and moisture content have been in use for some time, and a review of the early work has been given by Loos [119]. Now, densitometry and dendrochronology of tree cores are done routinely using scanning gamma transmission gauges, employing beam collimation widths as small as 0.1 mm to allow study of shapes as well as spacing of individual annual growth rings (e.g., Cown and Clement [120]). A somewhat different technique is described by Kouris et al. [121] in which a film is placed in contact with a thin slice of wood, and a radiograph of ring patterns is obtained using x rays emitted from a source on the other side. The optical film density variations, according to Kouris et al. [121], can be related to chemical composition variations, as well as simple density variations. Liu et al. [122] and Olson et al. [123] have recently reviewed theoretical wood densitometry, including (1) [122] mass attenuation equations and (2) [123] optimal x-ray energy for wood density measurement. X-ray computed axial tomography (CAT), has also found its way into the forest industries, in a portable CAT device demonstrated and described by Onoe et al. [124] for measuring the water content and distribution in the annual rings of living trees, also for noninvasively examining the interiors of utility poles for deterioration.

In woodworking factory situations the wood byproducts and their physical characteristics are frequently of importance. For example, for measuring the moisture and density of bulk quantities of spruce chips, Korell et al. [125] have investigated gamma transmission gauges, and also gamma albedo gauges in the form of immersion probes, similar in geometry to the well-logging probes used in mineral exploration, inserted into barrels of chips.

## 7. Radiometric Gauging and Control in Medicine and in the Medical Industries

Concern over osteoporosis has resulted in the development of a variety of radiometric gauges for noninvasive measurement of bone mineral. Peppier and Mazess [126] and Mazess et al. [127] have developed a dual energy photon transmission method for measuring the total body bone mineral content as well as the total lean body mass. Smith et al. [128] have compared the accuracy of photon absorptiometry (or transmission) for local bone mineral measurements with that for neutron activation, and conclude that neutron activation offers somewhat better precision.

Photon scattering bone density gauges have been used in studies of osteoporosis and treatment effectiveness by Roberts et al. [129]. Assessments of dual energy Compton scatter densitometers, including the effects of multiple scattering in both the object of interest and the overlying material, have been made by Huddleston and Weaver [130] and by Huddleston et al. [131]. Bone densitometers using the ratio of coherent to Compton scattering have been described by Stalp and Mazess [132] and by Shukla et al. [133].

Among the more exotic medical radioisotopic metrologies is a widely-used technique for studying the vibrations in the inner ear in which to the basilar membrane is attached a small radioactive source whose emission energies are Doppler-shifted to alter their transmissions through a fixed Mossbauer absorber, a technique pioneered by Johnstone and Boyle [134]. Kliauga and Khanna [135] examined the dose rate delivered to the inner ear in the course of such measurements, including a theoretical analysis which depended heavily on plaque-source radiation field formulas and tables, including exponential attenuation and buildup factor, given by Hubbell et al. [136] and by Hubbell [137,138].

In the medical pharmaceutical industry, a gamma-ray attenuation technique is used for monitoring the packing uniformity of powders for compressed tablets and for filling capsules, as described by Woodhead et al. [139], Woodhead and Newton [140], and Charlton and Newton [141].

## 8. Summary

In summary, we see that radiometric gauging and control devices employ photons, charged-particle beams, and neutrons in a great variety of both routine and some very specialized tasks. Some tasks, such as thickness control in hot rolling and forming of steel, and observation of the contents and status of sealed pipes and vessels, benefit particularly from the noncontact, nonintrusive nature of radiometric gauging and control.

Many of the above examples of radiometric gauging techniques have been developed and/or exploited in developing countries. This trend will probably continue, with accompanying international health-safeguard studies and local legislation to minimize the risks inherent in radiation usage while optimizing the technical and economic benefits. In all countries, sophisticated microcomputer analysis will extract additional useful information from the output of such complex devices as bore-hole loggers with both gamma and neutron sources and albedo spectrometer-detectors, also making more complete use of secondary signals as projected by McMaster [142] for all nondestructive evaluation (NDE) devices. Due also to increasing computer capabilities and availability, computed tomography (CT) will find more use across the board in industrial, agricultural, and medical situations, providing structural image information as well as density, thickness, and other parameters given by present radiation transmission and scatter gauges (Gilboy [143], Reimers et al. [144], and Vetter et al. [145]). However, process control in steel rolling and other hot fabrications, also in the paper and fabric industries, will likely continue to be dominated by present radiometric techniques through the remainder of the 1990s. In general, as pointed out by Charlton [146], radiation metrology will continue to gain favor with instrument engineers who are finding that nucleonic instruments, for a variety of special measurement problems, possess advantages offered by no other type of instrumentation.
